# Hepatitis B Virus and *Plasmodium falciparum* Co-Infection Among Pregnant Women in Gabon: Prevalence, Biochemical Impact, and Antagonistic Replication Dynamics

**DOI:** 10.3390/v17121576

**Published:** 2025-12-02

**Authors:** Aude Sandrine Andeme Eyi, Ismaël Pierrick Mikelet Boussoukou, Serge Thierry Omouessi, Jean Alban Ondh Obame, Opheelia Makoyo Komba, Joel Fleury Djoba Siawaya, Bénédicte Ndeboko

**Affiliations:** 1 Laboratoire du Centre Hospitalier, Universitaire Mère-Enfant Fondation Jeanne Ebori, Libreville BP 20000, Gabon; eyiaude@gmail.com (A.S.A.E.); mikeletpierrick@gmail.com (I.P.M.B.); joel.djoba@gmail.com (J.F.D.S.); 2 Département de Physiologie, Faculté de Médecine, Université des Sciences de la Santé, Libreville BP 20000, Gabon; omouessithierry@gmail.com; 3 Département de SVT, Ecole Normale Supérieur, Libreville BP 20000, Gabon; ondhobame@gmail.com; 4 Service de Gynécologie du Centre Hospitalier, Universitaire Mère-Enfant Fondation Jeanne Ebori, Libreville BP 20000, Gabon; makoyokombaopheelia@gmail.com; 5 Département de Biologie Cellulaire & Moléculaire, Génétique, Faculté de Médecine, Université des Sciences de la Santé, Libreville BP 20000, Gabon

**Keywords:** Hepatitis B virus, *Plasmodium falciparum*, co-infection, pregnancy, liver function, viral–parasitic interaction, sub-Saharan Africa, maternal health

## Abstract

**Background:** Hepatitis B virus (HBV) and *Plasmodium falciparum* infections remain major public health concerns in sub-Saharan Africa, especially among pregnant women, who are particularly vulnerable due to physiological immunomodulation. While mono-infections are well documented, the burden and biological consequences of HBV–*P. falciparum* co-infection during pregnancy remain under-investigated in Gabon. **Aim:** To determine the prevalence, clinical relevance, and biochemical impact of HBV–*P. falciparum* co-infection among pregnant women in Libreville, Gabon, and to explore the interaction between viral and parasitic replication. **Methods:** A prospective cross-sectional study was conducted between May 2022 and May 2023 at the CHUME-FJE Laboratory in Libreville. Serum samples were tested for HBsAg using rapid diagnostic tests and ELISA confirmation; HBV surface antigen (HBsAg) levels were quantified by electrochemiluminescence (ECLIA). Parasitemia was assessed by rapid diagnostic test, microscopy, and the Lambaréné thick blood film method. Liver function parameters (ALT, AST, ALP, and GGT) were evaluated using an automated biochemistry analyzer. Statistical analysis included Mann–Whitney U tests, chi-square tests and Spearman’s rank correlation coefficient with significance set at *p* < 0.05. **Results:** Of the 222 pregnant women enrolled, HBV infection was detected in 9 cases (4.05%). Among these, 6 (2.7% of the study population) were mono-infected with HBV, while 3 (1.35%) were co-infected with *Plasmodium falciparum*. *P. falciparum* parasitemia was detected in 58 cases (26.1%). Biochemical profiles revealed elevated transaminases (AST) in HBV mono-infected women, while liver enzymes remained within normal ranges in co-infected individuals. Quantitative analyses demonstrated an inverse relationship between HBV surface antigen levels and *P. falciparum* parasitemia. This observation could suggest an antagonistic replication dynamic. However, the relationship was not statistically significant (Spearman’s ρ = −0.5, *p* = 0.67). **Conclusions:** HBV and *P. falciparum* co-infection occurs in a small but clinically relevant proportion of pregnant women in Gabon. The observed inverse replication pattern suggests a potential biological antagonism that may modulate disease severity. These findings although preliminary, could highlight the need for integrated screening and management strategies during pregnancy to improve maternal and fetal outcomes.

## 1. Introduction

Hepatitis B virus (HBV) infection remains a critical global health issue, particularly in low- and middle-income countries, where access to preventive and therapeutic interventions is limited. HBV primarily targets hepatocytes, leading to acute or chronic liver disease. Chronic HBV infection can progress to cirrhosis, hepatic decompensation, and hepatocellular carcinoma (HCC), contributing significantly to global liver-related morbidity and mortality. An estimated 2 billion individuals worldwide have been exposed to HBV, with approximately 296 million living with chronic infection as of 2020 [1,2]. Despite the availability of an effective vaccine and antiviral therapies, HBV continues to cause nearly 650,000 deaths annually, with Africa accounting for about 125,000 of these fatalities [3,4]. 

Perinatal transmission remains a major route of HBV spread, particularly in endemic regions. Infected mothers can transmit the virus to their newborns during childbirth, and due to the immaturity of the neonatal immune system, up to 90% of exposed neonates may develop chronic HBV infection [5]. Preventing vertical transmission through systematic maternal screening and timely neonatal immunization is therefore a cornerstone of HBV control strategies.

In parallel, *Plasmodium falciparum* infection during pregnancy poses a significant threat to maternal and fetal health in sub-Saharan Africa. Malaria in pregnancy is associated with increased risks of maternal anemia, placental insufficiency, intrauterine growth restriction, preterm birth, and perinatal mortality. The immunological changes induced by pregnancy may exacerbate both malaria and HBV disease progression. Co-infection with HBV and *P. falciparum* has been linked to heightened hepatic inflammation, impaired immune response, and worsened clinical outcomes, including severe anemia, liver dysfunction, and adverse perinatal events [6,7,8,9].

Despite the overlapping endemicity of HBV and malaria in many African settings, including Gabon, limited data are available on the prevalence and clinical impact of HBV–*P. falciparum* co-infection during pregnancy. Addressing this gap is essential to informing public health strategies aimed at improving maternal and neonatal outcomes.

Therefore, the objective of this study was to assess the prevalence and potential clinical consequences of HBV and *Plasmodium falciparum* co-infection among pregnant women in Libreville, Gabon.

## 2. Materials and Methods

### 2.1. Study Site

This study was conducted at the laboratory of the Mother–Child University Hospital Center, Fondation Jeanne Ebori (CHUMEFJE), located in Libreville, the capital city of Gabon. CHUMEFJE is one of the four main university hospital centers in the country, and its laboratory comprises several specialized units enabling comprehensive diagnostic investigations.

### 2.2. Study Design and Population

A prospective, descriptive study was performed from 27 May 2022 to 30 May 2023. It involved plasma and serum samples obtained from pregnant women attending CHUMEFJE for routine antenatal care or other clinical consultations, whether pregnancy-related or not. Eligible participants were aged between 15 and 45 years and provided written informed consent prior to inclusion.

Blood samples were collected in both dry tubes (for serum separation) and EDTA tubes (for whole blood analysis). Serum samples were used for serological testing, while plasma and whole blood were used for malaria diagnosis. A total of 222 pregnant women were recruited, among whom three were identified as co-infected with both *Plasmodium falciparum* and hepatitis B virus (HBV).

### 2.3. Sample Size Consideration

This study was designed as a preliminary investigation. Therefore, no a priori power calculation was performed. Instead, all eligible pregnant women attending antenatal consultations during the study period and meeting the inclusion criteria were consecutively enrolled. The absence of a formal sample size calculation is acknowledged as a limitation, and the results should be interpreted accordingly.

### 2.4. Recruitment and Sample Collection

Participants were recruited through two channels:

1. Pregnant women attending the CHUMEFJE laboratory for blood collection were directly invited to participate and underwent sample collection on-site.
2. Additional participants were identified retrospectively through emergency hematology and gynecology registries as part of malaria-related hospitalizations. Relevant samples were collected and included in the study. In this case, it is important to specify that the daily circuit allowed us to catch pregnant women admitted for malaria; we thus collected their informed consent before including their sample in our study.

Demographic and clinical information were recorded using standardized data collection forms.

### 2.5. Serological Testing for HBsAg

Serum samples were screened for hepatitis B surface antigen (HBsAg) using the Determine™ HBsAg (ALERE, Abbott Point of Care, Abbott Park, IL, USA), rapid diagnostic test (RDT), which has a reported sensitivity of 96.4% and specificity of 100%. All RDT results—both positive and negative—were confirmed using the miniVIDAS^®^ ELISA system (Biomérieux, Lyon, France) following the manufacturer’s protocol.

### 2.6. Detection of Plasmodium falciparum

Malaria screening was conducted using whole blood collected in EDTA tubes. Initial screening was performed using malaria RDTs to detect *Plasmodium* antigens. Confirmatory testing was carried out via microscopy using a thick blood smear technique optimized in Lambaréné, Gabon. Briefly, 10 µL of whole blood were spread onto a 1.0 × 1.8 cm area on a clean glass slide. After air drying, the smear was stained with Giemsa for 15 min, rinsed with clean water, and allowed to dry. Slides were examined under oil immersion at 100× magnification.

### 2.7. Biochemical Analysis

Liver function tests, specifically alanine aminotransferase (ALT) and aspartate aminotransferase (AST), were measured using the Mindray BS200 automated analyzer (Mindray, Shenzhen, China). For each test, 400 µL of patient serum were placed into hemolysis tubes and loaded into the analyzer as per the machine’s interface instructions. Results were generated within approximately 20 min. Importantly, these tests were performed in all HBV-positive women (6 mono-infected and 3 co-infected), as well as in 6 randomly selected uninfected women and 6 P. falciparum mono-infected women, to allow subgroup comparisons.

### 2.8. Quantification of HBsAg

Quantitative analysis of HBsAg was performed using the Roche Cobas^®^ e411 immunoassay system (Roche Diagnostics, Mannheim, Germany). A volume of 500 µL of serum was transferred into a sample tube and loaded into the analyzer. The specific HBsAg quantification assay was selected via the system interface. The results were obtained after 45 min, following standard operating procedures. In addition, according to international clinical guidelines, HBV activity was based on both HBsAg quantification and liver enzyme (ALT) levels. Thus, HBsAg levels > 1000 IU/mL are suggestive of active hepatitis B infection, in accordance with these guidelines [10].

### 2.9. Statistical Analysis

Categorical variables were summarized as frequencies and percentages. Comparisons between groups (e.g., HBV mono-infected vs. HBV–*Plasmodium falciparum* co-infected) were made using non-parametric tests. Specifically, the Mann–Whitney U test was used to compare the quantitative HBsAg levels between the two groups. The chi-square (χ^2^) test or Fisher’s exact test was applied, as appropriate, to assess associations between categorical variables, such as the presence of active hepatitis B infection and co-infection status. A *p*-value < 0.05 was considered statistically significant. In addition, the relationship between HBsAg levels and *Plasmodium falciparum* parasitemia in co-infected individuals was assessed using Spearman’s rank correlation coefficient, a non-parametric test suitable for small sample sizes and non-normally distributed data. A two-tailed *p*-value < 0.05 was considered statistically significant. Graphs and figures were generated using GraphPad Prism version 9.0 (GraphPad Software, San Diego, CA, USA).

### 2.10. Ethical Considerations

The study protocol was reviewed and approved by the General Management and Scientific Council of CHUME-FJE (Approval ID: CHUME-FJE/008/22/05/20). All participants were fully informed about the study’s purpose, procedures, potential risks, and benefits. Written informed consent was obtained from each participant prior to enrollment, in the presence of a family witness when applicable. The study was conducted in accordance with the ethical principles outlined in the Declaration of Helsinki (1964) ( By The World Medical Association (WMA), Helsinki, Finland, June 1964, [11] and its subsequent revisions, as well as relevant national and institutional guidelines. Participant anonymity and confidentiality of data were strictly maintained throughout the research process.

## 3. Results

### 3.1. Prevalence of HBV and Plasmodium Falciparum Infections

**Figure 1:**
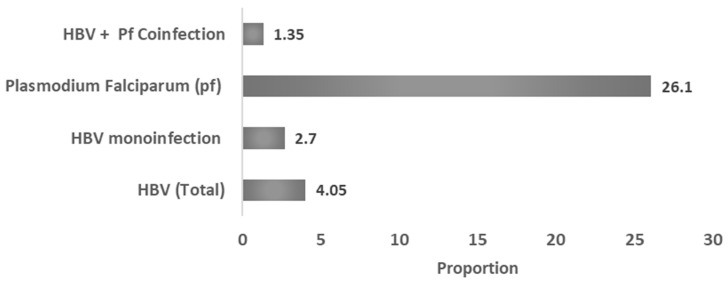
Prevalence of hepatitis B virus (HBV), *Plasmodium falciparum*, and co-infection among pregnant women (n = 222). A total of 9 women (4.05 %) tested positive for HBV, including 6 (2.7 %) with HBV mono-infection and 3 (1.35 %) co-infected with *P. falciparum*. Overall, *P. falciparum* infection was detected in 58 women (26.1 %). Percentages are expressed relative to the total study population.

A total of 222 pregnant women were enrolled in this study. Overall, 9 women (4.05%) tested positive for hepatitis B virus (HBV). Among them, 6 (2.7% of the study population) were mono-infected with HBV, while 3 (1.35%) were co-infected with *Plasmodium falciparum*. Independently, *P. falciparum* infection was detected in 58 women, corresponding to a prevalence of 26.1% (Figure 1).

### 3.2. Sociodemographic Parameters of Pregnant Women

#### 3.2.1. Age

The majority of HBV(+) women and co-infected women are in the 25–35 age group (77.7% and 33.3%, respectively). The Pf(+) group has a broader age distribution, with 41.3% under 25. The age group distribution differs across infection statuses, with potential significance according to χ^2^ or Fisher test.

#### 3.2.2. Residence

Most HBV(+) and co-infected women live in moderately developed neighborhoods. In contrast, a large proportion of Pf(+) women reside in underdeveloped neighborhoods (56.9%). Thus, residence may be significantly associated with *P. falciparum* infection risk, possibly due to environmental and vector exposure.

#### 3.2.3. Education Level

Among Pf(+) women, 43.1% are students, and 43.1% report having no formal education. The HBV(+) and co-infected groups include a higher proportion of active women. Therefore, educational attainment may be unevenly distributed.

#### 3.2.4. Marital Status

A high proportion of single women is noted in all groups, particularly Pf(+) (91.3%). Co-infected women show a mix, with 66.6% single and 33.3% married. Thus, marital status may influence exposure risk via sociobehavioral factors.

Taken together, the analysis reveals statistically significant associations between infection status and sociodemographic factors, particularly age group and residence. These findings highlight the potential role of environmental and demographic variables in infection risk among pregnant women (Table 1).

### 3.3. Biochemical Parameters and Group Stratification

To evaluate hepatic function in the context of mono-infection and co-infection, patients were categorized into four groups:

Group 1: Uninfected (HBV−/Pf−)

Group 2: HBV mono-infection (HBV+/Pf−)

Group 3: *P. falciparum* mono-infection (HBV−/Pf+)

Group 4: HBV and *P. falciparum* co-infection (HBV+/Pf+)

Liver function was assessed in a representative subset of six patients from each group using serum biochemical markers, including alanine aminotransferase (ALT), aspartate aminotransferase (AST), gamma-glutamyl transferase (GGT), alkaline phosphatase (ALP), and total bilirubin.

Group 1 (HBV−/Pf−): All patients exhibited transaminase levels within normal ranges. One patient showed a slight elevation in ALP, which may be attributable to physiological changes during pregnancy.

Group 2 (HBV+/Pf−): All patients demonstrated markedly elevated AST levels, suggesting hepatocellular injury associated with HBV infection. Other liver enzymes (ALT, GGT, and ALP) remained within normal limits in most cases. However, two patients in this group exhibited abnormally high GGT and ALP levels, indicative of significant hepatic impairment.

Group 3 (HBV−/Pf+): With the exception of one patient showing minor transaminase alterations, the majority of patients had liver enzyme values within normal reference ranges. This suggests that isolated *P. falciparum* infection did not consistently cause liver dysfunction in the studied cohort.

Group 4 (HBV+/Pf+): All co-infected patients had normal transaminase levels. No biochemical evidence of exacerbated hepatic dysfunction was observed in this group, although the sample size was limited.

### 3.4. Quantification of Hepatitis B Surface Antigen (HBsAg) in Patients Infected with Hepatitis B Virus

**Figure 2:**
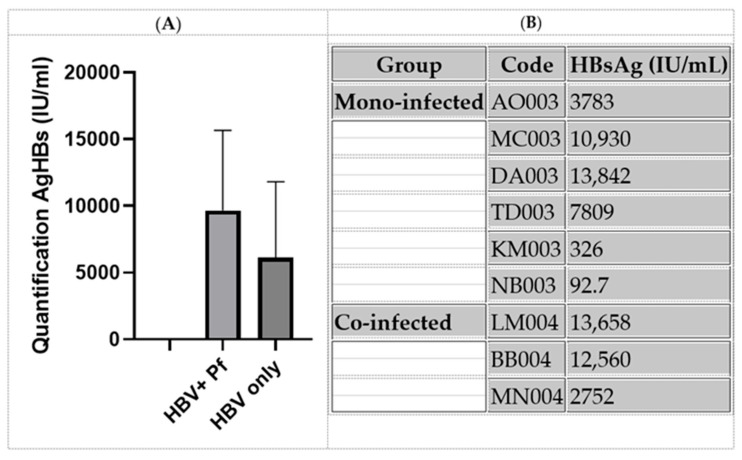
Quantification of hepatitis B surface antigen (HBsAg) in HBV mono-infected and HBV + *P. falciparum* co-infected individuals (**A**,**B**).

Quantification of HBsAg revealed a broad range of antigen levels among HBV–infected individuals. The median HBsAg level in mono-infected individuals was lower compared to those co-infected with *Plasmodium falciparum*, although the difference was not statistically significant (Mann–Whitney U test, *p* > 0.05) (Figure 2).

According to international clinical guidelines, HBsAg levels > 1000 IU/mL are suggestive of active hepatitis B infection. Among co-infected individuals, two out of three (LM004 and BB004) had elevated antigen levels (>1000 IU/mL), suggesting active replication, while one (MN004) remained below this threshold (Figure 2).

In addition, a striking observation was that in patients co-infected with HBV and *Plasmodium falciparum*, there appeared to be an inverse relationship between viral antigen levels and parasitemia. For instance, MN004 exhibited the lowest HBsAg value (2752 IU/mL) and the highest (11,560 P/μL), whereas LM004 and BB004 had elevated HBsAg levels and relatively lower parasitemia. These findings suggest a possible competitive or antagonistic interaction between HBV replication and *Plasmodium falciparum* proliferation (Table 2).

### 3.5. Hepatitis B Virus Infection Activity in Pregnant Women

**Figure 3:**
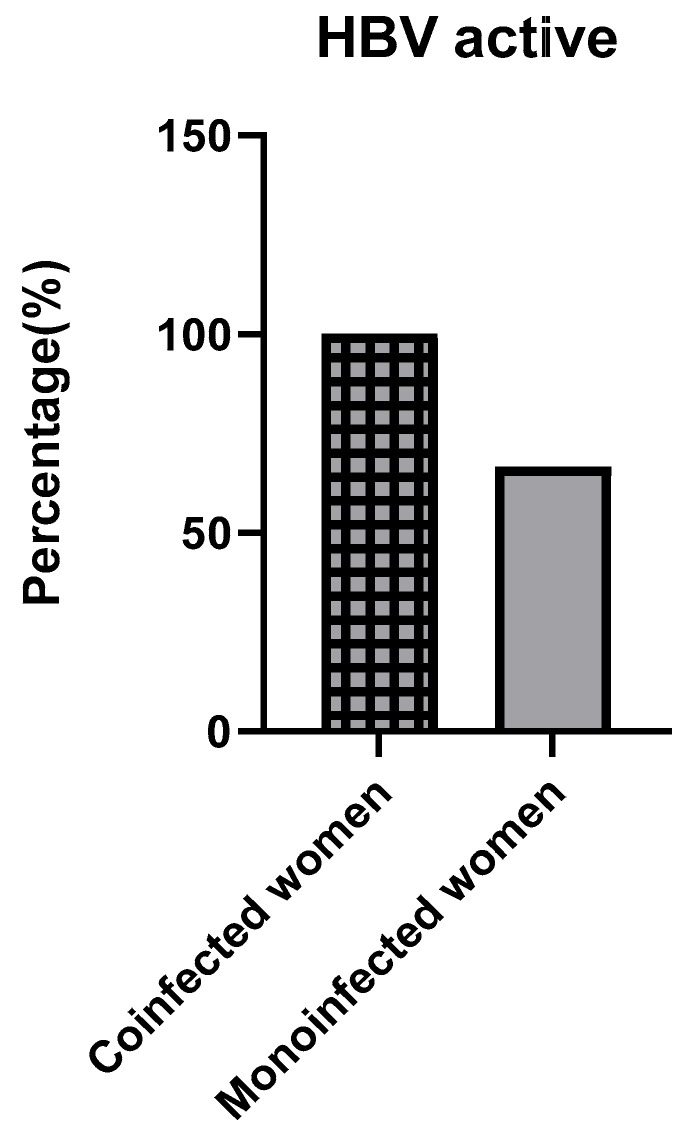
Proportion of active HBV infection among mono-infected vs. co-infected pregnant women.

Among the pregnant women infected with hepatitis B virus, 6 were mono-infected and 3 were co-infected with *Plasmodium falciparum*. Active hepatitis B infection, defined by HBsAg levels > 1000 IU/mL, was observed in 4 out of 6 (66%) mono-infected women. Interestingly, all co-infected pregnant women (3/3, 100%) had active HBV infection. This trend reinforces the observation that co-infection may enhance HBV replication in certain immunological contexts, though it is also consistent with the inverse relationship seen in individual quantification data (Figure 3).

## 4. Discussion

The current study provides new insights into the epidemiological and virological profile of hepatitis B virus (HBV) infection in women, with a particular focus on atypical serological profiles and the potential impact of co-infection with *Plasmodium falciparum*. The integration of molecular data through quantitative HBsAg (qHBsAg) testing has enabled a more accurate characterization of HBV replication activity, particularly in seronegative or atypical cases.

### 4.1. HBV Replication Activity and qHBsAg Levels

Our data demonstrate that the majority of patients with high qHBsAg levels (>1000 IU/mL) exhibit active HBV infection, consistent with prior studies indicating that qHBsAg can serve as a surrogate marker for intrahepatic covalently closed circular DNA (cccDNA) and viral replication [12,13]. In this context, qHBsAg was a valuable marker in identifying active infection even among patients who were negative for HBeAg or showed atypical serological patterns.

The detection of active infection in the absence of HBeAg supports the hypothesis of pre-core or basal core promoter mutations, which have been frequently reported in West African populations [14]. This finding justifies the routine use of qHBsAg, especially in regions where occult or mutant HBV forms may be underdiagnosed using conventional serological assays.

### 4.2. HBV–Plasmodium falciparum Interaction

A particularly novel finding of our study is the possible antagonistic interaction between HBV replication and *Plasmodium falciparum* parasitemia. Indeed, among co-infected patients, we observed an inverse relationship between HBsAg levels and parasite density. For instance, one patient with high parasitemia (11,560 P/μL) exhibited relatively low HBsAg levels (2752 IU/mL), whereas two patients with low-to-moderate parasitemia had markedly elevated HBsAg values (>12,000 IU/mL). These observations support previous hypotheses suggesting a competitive interplay between hepatotropic and hemotropic pathogens within the hepatic microenvironment [15,16].

Statistically, Spearman’s rank correlation analysis revealed a moderate inverse relationship between HBsAg levels and *P. falciparum* parasitemia (ρ = −0.5, *p* = 0.67), but it was not statistically significant.

This inverse correlation may be explained by immunological modulation or competition for hepatic cellular machinery. Some reports suggest that malaria-related immune activation may transiently suppress HBV replication, while others propose that HBV–induced alterations in liver immunity may influence parasite sequestration or clearance [6].

### 4.3. Impact on Pregnant Women

Of particular concern is the observation that all co-infected pregnant women (3/3, 100%) displayed active HBV replication, compared to 66% (4/6) among mono-infected women. While the sample size remains limited, this suggests a potential synergy between pregnancy-related immune modulation and co-infection, favoring viral replication. Pregnancy is known to alter innate and adaptive immune responses, potentially reducing viral clearance [17]. The presence of *Plasmodium falciparum* may further exacerbate this state by inducing inflammatory pathways or disrupting hepatic antigen presentation.

Given the elevated risk of vertical transmission in women with high qHBsAg levels, our findings underscore the need for targeted antiviral prophylaxis in pregnant women, particularly those with evidence of co-infection or high HBsAg titers, in accordance with WHO guidelines [10,18]. Importantly, HbsAg quantification should be tested to characterize disease phase, define prognosis, and guide treatment as HBeAg according to the new EASL Clinical Practice Guidelines on the management of hepatitis B virus infection [19].

### 4.4. Clinical Implications

The combined use of qHBsAg quantification and parasite load measurement provides a promising approach for stratifying HBV–infected patients in endemic areas. Moreover, these preliminary results emphasize the necessity of integrated screening and treatment programs for both HBV and malaria, particularly in women of reproductive age.

### 4.5. Limitations of This Study

A limitation of this study is the small sample size and the absence of a priori power calculation. As this was a preliminary investigation, the sample size was determined by the availability of participants rather than statistical power considerations. Consequently, the findings, although interesting, should be interpreted with caution and confirmed in larger, adequately powered studies.

## 5. Conclusions

This study highlights the occurrence and biological implications of HBV and *Plasmodium falciparum* co-infection among pregnant women in Libreville, Gabon. Although the co-infection prevalence was low, its clinical relevance is underscored by distinct biochemical patterns and an apparent inverse interaction between viral and parasitic replication. The reduced liver enzyme levels in co-infected individuals, along with the observed antagonism between HBV replication and parasitemia, suggest a complex interplay that may aggravate liver injury during co-infection. These preliminary results, which appear interesting, should be confirmed in a study with a larger cohort. Indeed, such observations could highlight the importance of taking co-infections into account in maternal health care protocols and advocate for integrated diagnostic and surveillance approaches to optimize antenatal care in endemic regions.

## Figures and Tables

**Table 1:** Sociodemographic Parameters of Pregnant Women.

Characteristic	HBV Mono-Infected (n = 6)	Pf Mono-Infected (n = 55)	Co-Infected (n = 3)	*p*-Value (Fisher)
Age group (years)				0.063
<25	0 (0.0%)	23 (41.8%)	1 (33.3%)	
25–35	6 (100%)	29 (52.7%)	1 (33.3%)	
>35	0 (0.0%)	3 (5.5%)	1 (33.3%)	
Residence				0.063
Developed neighborhood	1 (16.7%)	6 (10.9%)	0 (0%)	
Moderately developed	5 (83.3%)	17 (30.9%)	2 (66.7%)	
Underdeveloped neighborhood	0 (0.0%)	32 (58.2%)	1 (33.3%)	
Education level				0.213
Student	2 (33.3%)	24 (43.6%)	1 (33.3%)	
Active women	4 (66.7%)	16 (29.1%)	2 (66.7%)	
None	0 (0%)	15 (27.3%)	0 (0%)	
Marital status				0.261
Single	5 (83.3%)	51 (92.7%)	2 (66.7%)	
Married	1 (16.7%)	4 (7.3%)	1 (33.3%)	

**Table 2:** HBsAg and parasitemia levels.

Patient Code	HBsAg (IU/mL)	Parasitemia (P/μL)
LM004	13,658	6160
BB004	12,560	3850
MN004	2752	11,560

Spearman’s rank correlation analysis revealed a moderate inverse relationship between HBsAg levels and *P. falciparum* parasitemia (ρ = −0.5, *p* = 0.67). Although not statistically significant due to the small sample size, this trend supports the hypothesis of potential antagonistic replication dynamics between the two pathogens.

## Data Availability

The original contributions presented in this study are included in the article/supplementary material. Further inquiries can be directed to the corresponding author.
